# Crosstalk Between DNA and Histones: Tet’s New Role in Embryonic Stem Cells

**DOI:** 10.2174/138920212803759730

**Published:** 2012-12

**Authors:** Xinyi Sui, Colles Price, Zejuan Li, Jianjun Chen

**Affiliations:** 1 Section of Hematology/Oncology, Department of Medicine; 2 The College, University of Chicago, Chicago, IL 60637, USA

**Keywords:** Tet, ES cells, Polycomb repressive complex, DNA modification, Histone modification, Epigenetics, 5mC, 5hmC.

## Abstract

Embryonic stem (ES) cells are characterized by the expression of an extensive and interconnected network of pluripotency factors which are downregulated in specialized cells. Epigenetic mechanisms, including DNA methylation and histone modifications, are also important in maintaining this pluripotency program in ES cells and in guiding correct differentiation of the developing embryo. Methylation of the cytosine base of DNA blocks gene expression in all cell types and further modifications of methylated cytosine have recently been discovered. These new modifications, putative intermediates in a pathway to erase DNA methylation marks, are catalyzed by the ten-eleven translocation (Tet) proteins, specifically by Tet1 and Tet2 in ES cells. Surprisingly, Tet1 shows repressive along with active effects on gene expression depending on its distribution throughout the genome and co-localization with Polycomb Repressive Complex 2 (PRC2). PRC2 di- and tri-methylates lysine 27 of histone 3 (H3K27me2/3 activity), marking genes for repression. In ES cells, almost all gene loci containing the repressive H3K27me3 modification also bear the active H3K4me3 modification, creating “bivalent domains” which mark important developmental regulators for timely activation. Incorporation of Tet1 into the bivalent domain paradigm is a new and exciting development in the epigenetics field, and the ramifications of this novel crosstalk between DNA and histone modifications need to be further investigated. This knowledge would aid reprogramming of specialized cells back into pluripotent stem cells and advance understanding of epigenetic perturbations in cancer.

## INTRODUCTION

Embryonic stem (ES) cells are isolated from the inner cell mass of the early-stage blastocyst and can be characterized by their pluripotency and capacity for self-renewal. The pluripotent stem cell program is driven by the “core” transcription factors Oct4, Sox2 and Nanog which form a stable autoregulatory loop that can also poise the cell for differentiation [1]. The pluripotent state also displays characteristic epigenetic marks, including bivalent histone domains on developmental regulators [2] and prominent non-CpG methylation throughout the genome [3]. 

Histone modifications can be either activating or repressive depending on the location and the chemical mark. Two canonical modifications are trimethylation at lysine 27 of histone H3 (H3K27me3), which is a repressive mark catalyzed by Polycomb Repressive Complex 2 (PRC2), and trimethylation at lysine 4 of histone H3 (H3K4me3), which is an activating mark catalyzed by the Trithorax family of proteins [4]. H3K27me3 and H3K4me3 co-localize on a significant number of developmental regulators in ES cells, forming the distinctive “bivalent domain” which poises these gene loci for activation upon differentiation [5].

DNA methylation was thought to be responsible for long-term and stable repression of gene expression and has been implicated in X-inactivation, genomic imprinting and retrotransposon silencing [6]. In adult somatic cells, DNA methylation and the resultant 5-methylcytosine (5mC) mark predominantly occur at the cytosine of CpG dinucleotides, but in ES cells near a quarter of all DNA methylation happens in a non-CpG context [3]. Recently, global demethylation events have been characterized in the zygote and developing primordial germ cells [7-10], and extensive turnover of loci-specific methylation has been observed during cellular differentiation [3, 11], changing our conception of DNA methylation into that of a more dynamic mark. 

The discovery of the ten-eleven translocation (Tet) family of DNA hydroxylases revolutionized the epigenetics field, sparking an incredibly rapid investigation of possible DNA demethylation pathways mediated by Tet and of the protein family’s functional significance in multiple important gene regulation contexts, including cancer and ES cells. There are three members within the Tet family of proteins, namly Tet1, Tet2 and Tet3, and this review will primarily focus on the first two members, which are upregulated in the pluripotent state and downregulated during differentiation [12]. While many details about the regulation of DNA methylation are still unknown, an exciting discovery involves the novel dual functions of Tet1 occupancy on specific gene loci in the ES cell [13-15]. These recent studies link DNA modification status with that of the surrounding histone marks, and this crosstalk is important to keep in mind for future manipulations of gene regulation, whether in the context of induced pluripotent stem (iPS) cells or cancer treatment.

## ROLE OF TET IN DNA DEMETHYLATION

Tet1 is a Fe(II)- and alpha-ketoglutarate (α-KG)-dependent DNA hydroxylase that was first discovered as a fusion partner of the mixed lineage leukemia (*MLL*) gene in a rare form of acute myeloid leukemia (AML) containing the t(10; 11)(q22;q23) translocation [16, 17]. Tet1 and Tet2, as well as Tet3, became subjects of intense research once they were found to have catalytic activity on 5-methylcytosine (5mC) [18], converting the initial cytosine modification to 5-hydroxymethylcytosine (5hmC), 5-formylcytosine (5fC) and 5-carboxylcytosine (5caC) through successive oxidation steps [10, 19-21]. 5hmC accumulates in cells at appreciable levels of around a hundred to several thousand modified bases per million unmodified C, but 5fC and 5caC levels are barely detectable, even in cell types enriched with the Tet family of proteins [21]. However, accumulation of 5caC levels can be observed when thymine-DNA glycosylase, responsible for excision of 5caC, is depleted in mouse embryonic stem cells [21].

These new cytosine modifications are widely believed to be the first steps of active DNA demethylation, but there has only been one study showing a robust mechanism for replication-independent demethylation [22]. There are currently three theories about how active demethylation can be completed from Tet-mediated cytosine modifications [23] see Fig. (**1**). First, 5fC or 5caC could be excised by thymine DNA glycosylase (TDG) [20] and subsequently replaced with unmodified C through the Base Excision Repair (BER) pathway. Second, 5hmC could be deaminated by the activation induced deaminase/apolipoprotein B editing complex (AID/APOBEC) family of cytidine deaminases to form 5-hydroxymethyluracil (5hmU), which would then also be excised by a DNA glycosylase and subject to BER [24]. Third, 5caC could be directly decarboxylated to generate unmodified cytosine, though there is yet no direct evidence of decarboxylase. The second mechanism involving AID/ APOBEC and mediated by Tet1 has been observed *in vivo *in the adult mouse brain, though the research group did not rule out the presence of other contributing mechanisms [22]. AID deficiency also weakens global demethylation in mouse primordial germ cells [25]. However, the two BER pathways are considered unlikely to be solely responsible for the global demethylation events seen in embryonic development, as large-scale BER would put excessive strain on genome stability [26]. Furthermore, a recent study has revealed no detectable deamination of 5hmC by AID/APOBEC *in vitro*, which the authors attribute to 5hmC’s increased steric bulk compared to cytosine and 5mC [27]. This study challenges the viability of the AID/APOBEC demethylation pathway. The search for a 5caC decarboxylase – or another yet-undiscovered mechanism for genome-wide demethylation - will undoubtedly continue in the future.

A pathway for passive demethylation using 5hmC has also been elucidated. On the basis of findings from others that Tet3-mediated oxidation of 5mC to 5hmC is important for global demethylation of the paternal genome in zygotes and preimplantation embryos [28-30], Inoue *et al.* suggested that loss of 5hmC during preimplantation is likely a DNA replication-dependent passive process by use of an immunostaining approach [31, 32]. Nonetheless, caution should be exercised, because immunostaining is not quantitative and therefore it is possible that only a portion of 5mC is converted to 5hmC whereas the remaining portion of 5hmC is removed by an alternative pathway [33].

## ROLE OF TET IN EMBRYONIC STEM CELLS

The role of 5hmC and the Tet family of proteins in ES cell pluripotency, self-renewal and lineage specification has been discussed ever since the function of Tet1 was first discovered [18]. Both Tet1 and Tet2 are upregulated in ES cells – though Tet1 shows up to 5-fold higher expression than Tet2 – and their expression levels drop after induced differentiation; similarly, 5hmC is also enriched in ES cells and downregulated during development [18, 34, 35]. Tet1 and Tet2 have been shown to be binding targets of Oct4 [34], incorporating the proteins into well-characterized pluripotency machinery, but the necessity of Tet1/2 to pluripotency maintenance is still unclear. Several groups have shown significant loss of ES cell morphology after Tet1 knockdown, coinciding with a decrease in 5hmC levels and downregulation of Nanog through hypermethylation of its promoter [12, 18]. Other groups have observed bias in lineage specification after knockdown of Tet1 or Tet2, skewing ES cells towards trophoectoderm or mesoendoderm commitment by upregulating specific differentiation genes such as Cdx2, Gata6, Eomes and Elf5 [12, 34]. Interestingly, Koh *et al.* found that Tet1 and Tet2 showed antagonistic effects against each other at several developmental regulators, suggesting that, despite having similar function, the proteins may be responsible for two different lineage specifications [34]. Koh *et al.* [34] also saw no effect on Nanog expression or the stem cell phenotype after Tet1 or Tet2 knockdown, an observation that was verified by other groups [15]. Similarly, Dawlaty *et al.* [36] were able to generate Tet1 knockout ES cells that retained pluripotency and in fact developed into knockout pups. They also generated viable Tet1 knockout mice through crosses of heterozygous Tet1^+/-^ mice, though the mutant progeny were slightly smaller in body size than wild-type mice [36]. It is possible that the effects of Tet1 knockout were compensated for by Tet2 activity, and it will be important to generate double knockout ES cells and mice in order to fully test for the role of Tet proteins in pluripotency. 

The molecular mechanisms behind Tet1 and Tet2’s functions in ES cells are not clearly proven, though, surprisingly, demethylation of bound loci is not the only effect of Tet1 occupancy. Many genome-wide studies of Tet, 5mC and 5hmC occupancy have been conducted in order to better understand the functions and interactions of these epigenetic marks [13, 15, 23, 37-42], and new techniques have been created to detect 5hmC on genome-wide scale [43, 44] and more recently also at single-base resolution [45-47]. 

A majority of the genome-wide studies discovered new complexities in Tet1 and 5hmC signaling in ES cells. First, Tet1 and 5hmC did not colocalize as extensively as would be expected from their association, though both were clustered in gene-rich areas of the genome [15, 26, 37]. Tet1 binds DNA through its N terminus CXXC domain, which has been shown to bind preferentially to unmodified, CpG rich DNA [15]. In light of this binding preference, it is not surprising that Tet1 is heavily enriched at high CpG promoters and exons [15, 26, 37], which have been previously associated with low DNA methylation [48]. However, 5hmC was shown to be excluded from high CpG density promoters, even though the mark is enriched according to CpG density within the gene body [15]. One group found only a 30% overlap of Tet1 and 5hmC peaks within the ES genome, distributed roughly equally between promoter, intron, exon and intergenic regions [15]. At these overlapping regions, Tet1 knockdown downregulates 5hmC levels and upregulates 5mC levels, but Tet1 knockdown does not affect 5hmC levels at other gene loci [15]. The disparity between Tet1 and 5hmC loci does not, by itself, prove a new function for either Tet1 or 5hmC. Tet1 could be acting on 5mC without fully binding to the DNA, and 5hmC could be too transient an intermediate between 5mC and further oxidation derivatives to be detected at Tet1-bound loci. However, when looking for differential gene expression between control and Tet1-knockdown cells, researchers observed both Tet1-activated and Tet1-repressed targets, direct evidence for a novel repressive function of Tet1 [13-15, 26]. In fact, both groups observed a greater number of Tet1-repressed targets, i.e. genes that are upregulated upon Tet1 knockdown, than Tet1-activated targets, or genes that are downregulated upon Tet1 knockdown [13-15, 26]. This finding, combined with other evidence, suggested a new mechanism of gene expression by Tet1 outside of hydroxymethylation. 

## REGULATION OF iPS CELLS BY TET2

An intriguing area of interest is the study of induced pluripotent stem cells (iPS) and the ability of these cells to be used in science and medicine. These cells are generated by reprogramming somatic cells by using pluripotency factors Oct4, Sox2, Klf4 and c-MYC (referred to as OSKM) [49]. Very recently, Doege *et al.* found reprogramming by OSKM led to Parp1 (ADP-ribose polymerase-1) and Tet2 recruitment to the loci of *Nanog* and *Esrrb*, previously known pluripotency loci [50]. Although somatic epigenetic signatures are lost in iPSC reprogramming [51] and both genes were previously indicated in epigenetic remodeling, this was the first evidence for their role in the reprogramming of pluripotency loci. They found that Tet2, but not Tet1 or Tet3, was significantly induced in somatic cells transformed with OSKM both at early reprogramming stages and at the iPSC stage. Moreover, loss of Parp1 or Tet2 led to decreased chromatin-active histone modifications at pluripotency loci. Reduction of Tet2 blocked induction of hydroxymethylation at pluripotency loci while methylation changes varied. This, combined with the early induction of hydroxymethylation at these loci, suggests that 5hmC potentially serves as a distinct epigenetic mark from 5mC. Together these results reveal a new function of Tet2 in the induction of pluripotency as well as suggesting that hydroxymethylation potentially acts outside the demethylation pathway and might promote chromatin remodeling [50].

## THE POLYCOMB REPRESSIVE COMPLEX IN EMBRYONIC STEM CELLS

Another key component to the control of ES cell differentiation is the regulation of the Polycomb Repressive Complexes 1 and 2 (i.e., PRC1 and PRC2), with a majority of the existing scientific research focused on PRC2. The PRC1/2 complexes create repressive histone marks on their target genes, thus blocking expression of these genes. The repressive marks known to be catalyzed by PRC1 and PRC2, to date, are monoubiquitylation of histone H2AK119 (H2AK 119u1) and dimethylation and trimethylation of histone H3K27 [52-58]. While the Polycomb complexes have been known for several years to be important regulators of cell fate decision [59, 60], it was not until recently that this complex was clearly tied to the maintenance and differentiation of ES cells [61]. In 2006, several groups showed simultaneously that Polycomb regulated targets were transcriptionally repressed in both human and mouse ES cells and reactivated during ES cell differentiation [62-64]. Since then, more evidence has accumulated that highlights the importance of these complexes in the regulation of embryonic stem cells. For example, knockout of SUZ12, EZH2, PCL2, EED or JARID2, all components of PRC2, in embryonic stem cells results in the inability of these stem cells to undergo proper differentiation and the expression of higher levels of pluripotency markers (reviewed in [65]). Those studies have demonstrated that PRC1 and PRC2 are essential components of ES cell maintenance and differentiation.

## TET1 INTERACTION WITH THE POLYCOMB REPRESSIVE COMPLEX IN EMBRYONIC STEM CELLS

While it was known that both Tet1 expression and PRC1 and PRC2 regulation were required to maintain embryonic stem cells, there was little to no functional connection between Tet1 and PRC1/2 until very recently. In 2011, Wu *et al.* [13, 14, 23, 26] connected Tet1-repressed targets to the recruitment of a repressive histone mark, H3K27me3, to the gene promoter. They also discovered that Tet1 knockdown substantially compromises the ability of Ezh2 to bind to PRC2/Tet1 cobound gene loci in ES cells [13, 14, 23, 26] see Fig. (**1**). Importantly, Nanog overexpression in Tet1-knock down cells cannot fully rescue Ezh2 binding to its target loci, showing that Tet1’s effects on Ezh2 do not result from disruption of core pluripotency machinery [13, 14, 23, 26]. No stable interactions have yet been observed between Tet1 and Ezh2, and Tet1 knockdown does not reduce PRC2 expression or stability [13, 14, 23, 26], producing an intriguing question – how does Tet1 affect PRC2 binding without direct interaction with its subunits?

## FUTURE WORK

There is still much we do not know about DNA modifications and about epigenetic regulation in general. New techniques have been developed to distinguish 5hmC from 5mC, even at single-base resolution [45-47], but these methods need to be further refined to be able to determine all layers of DNA modifications (5mC, 5hmC, 5caC and 5fC) at a single cell level. Once detection methods for all epigenetic marks and protein-DNA binding have reached this degree of resolution, we will be able to better understand how crosstalk interactions occur between epigenetic regulatory complexes and their resultant marks. 

More work is also required to better understand the connection between reading, writing and interpreting the histone code, both on its own and as it relates to other epigenetic factors including methylation/hydroxymethylation. Specifically in ES cells, a majority of the research so far has focused on the function and localization of Tet1, and Tet2 must be incorporated into this regulatory picture. Other epigenetic mechanisms should also be integrated, including the further oxidation derivatives of 5hmC, other histone modifications and ES cell-specific non-coding RNAs. This future work will provide a better understanding of stem cell pluripotency and of crosstalk between the different mechanisms of gene regulation.

## Figures and Tables

**Fig. (1):**
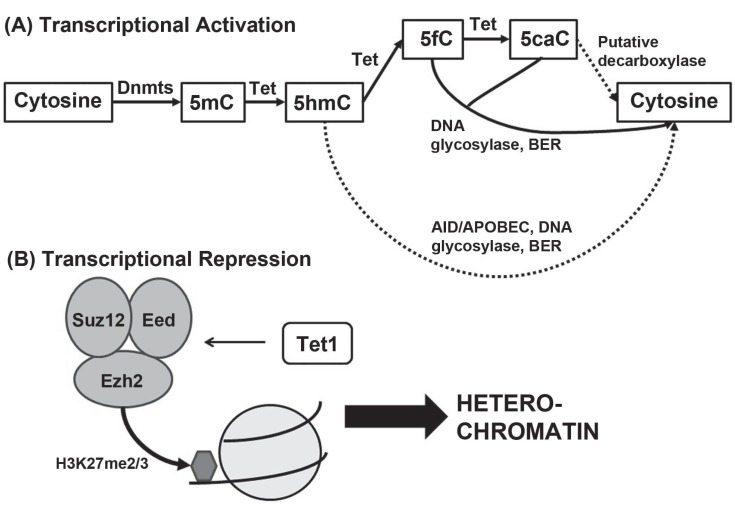
Dual roles of Tet1 in embryonic stem cells. (**A**) The conventionally recognized role of Tet1 as a transcriptional activator. Even though the pathways for active demethylation have not been fully elucidated, 5hmC, 5fC and 5caC are still generally accepted as intermediates in the demethylation pathway. (**B**) The novel role of Tet1 as a transcriptional repressor. Tet1 indirectly mediates binding of EZH2 to gene promoters to create the repressive H3K27me3 mark, leading to heterochromatin and repression of gene expression.

## ABBREVIATIONS

ES: = Embryonic Stem
Tet: = Ten-Eleven Translocation
PRC: = Polycomb Repressive Complex
H3K27me3: = Trimethylation At Lysine 27 of Histone H3
H3K4me3: = Trimethylation At Lysine 4 of Histone H3
5mC: = 5-Methylcytosine
5hmC: = 5-Hydroxymethylcytosine
5fC: = 5-Formylcytosine
5caC: = 5-Carboxylcytosine
iPS: = Pluripotent Stem
*MLL*: = Mixed Lineage Leukemia
AML: = Acute Myeloid Leukemia
TDG: = Thymine DNA Glycosylase
BER: = Base Excision Repair
AID: = Activation Induced Deaminase
APOBEC: = Apolipoprotein B Editing Complex

## References

[1] Young RA (2011). Control of the embryonic stem cell state. Cell.

[2] Boyer LA, Mathur D, Jaenisch R (2006). Molecular control of pluripotency. Current Opinion in Genetics & Development.

[3] Lister R, Pelizzola M, Dowen R H, Hawkins RD, Hon G, Tonti-Filippini J, Nery JR, Lee L, Ye Z, Ngo QM, Edsall L, Antosiewicz-Bourget J, Stewart R, Ruotti V, Millar AH, Thomson JA, Ren B, Ecker JR (2009). Human DNA methylomes at base resolution show widespread epigenomic differences. Nature.

[4] Schuettengruber B, Martinez A M, Iovino N, Cavalli G (2011). Trithorax group proteins: switching genes on and keeping them active. Nature reviews. Molecular Cell Biology.

[5] Zhao XD, Han X, Chew J L, Liu J, Chiu KP, Choo A, Orlov YL, Sung WK, Shahab A, Kuznetsov VA, Bourque G, Oh S, Ruan Y, Ng HH, Wei CL (2007). Whole-genome mapping of histone H3 Lys4 and 27 trimethylations reveals distinct genomic compartments in human embryonic stem cells. Cell Stem Cell.

[6] Bird A (2002). DNA methylation patterns and epigenetic memory. Genes & Development.

[7] Mayer W, Niveleau A, Walter J, Fundele R, Haaf T (2000). Demethylation of the zygotic paternal genome. Nature.

[8] Oswald J, Engemann S, Lane N, Mayer W, Olek A, Fundele R, Dean W, Reik W, Walter J (2000). Active demethylation of the paternal genome in the mouse zygote. Current Biology.

[9] Hajkova P, Erhardt S, Lane N, Haaf T, El-Maarri O, Reik W, Walter J, Surani MA (2002). Epigenetic reprogramming in mouse primordial germ cells. Mechanisms of Development.

[10] Sasaki H, Matsui Y (2008). Epigenetic events in mammalian germ-cell development: reprogramming and beyond. Nature Reviews Genetics.

[11] Meissner A, Mikkelsen TS, Gu H, Wernig M, Hanna J, Sivachenko A, Zhang X, Bernstein BE, Nusbaum C, Jaffe DB, Gnirke A, Jaenisch R, Lander ES (2008). Genome-scale DNA methylation maps of pluripotent and differentiated cells. Nature.

[12] Ito S, D'Alessio AC, Taranova OV, Hong K, Sowers LC, Zhang Y (2010). Role of Tet proteins in 5mC to 5hmC conversion, ES-cell self-renewal and inner cell mass specification. Nature.

[13] Wu H, D'Alessio AC, Ito S, Wang Z, Cui K, Zhao K, Sun YE, Zhang Y (2011). Genome-wide analysis of 5-hydroxy methyl cytosine distribution reveals its dual function in transcriptional regulation in mouse embryonic stem cells. Genes & Development.

[14] Wu H, D'Alessio AC, Ito S, Xia K, Wang Z, Cui K, Zhao K, Sun YE, Zhang Y (2011). Dual functions of Tet1 in transcriptional regulation in mouse embryonic stem cells. Nature.

[15] Xu Y, Wu F, Tan L, Kong L, Xiong L, Deng J, Barbera A J, Zheng L, Zhang H, Huang S, Min J, Nicholson T, Chen T, Xu G, Shi Y, Zhang K, Shi YG (2011). Genome-wide regulation of 5hmC, 5mC, and gene expression by Tet1 hydroxylase in mouse embryonic stem cells. Molecular Cell.

[16] Ono R, Taki T, Taketani T, Taniwaki M, Kobayashi H, Hayashi Y (2002). LCX leukemia-associated protein with a CXXC domain, is fused to MLL in acute myeloid leukemia with trilineage dysplasia having t(10,11)(q22,q23). Cancer Research.

[17] Lorsbach RB, Moore J, Mathew S, Raimondi SC, Mukatira ST, Downing JR (2003). TET1, a member of a novel protein family, is fused to MLL in acute myeloid leukemia containing the t(10,11)(q22,q23). Leukemia.

[18] Tahiliani M, Koh KP, Shen Y, Pastor WA, Bandukwala H, Brudno Y, Agarwal S, Iyer LM, Liu DR, Aravind L, Rao A (2009). Conversion of 5-methylcytosine to 5-hydroxymethylcytosine in mammalian DNA by MLL partner TET1. Science.

[19] Zhang H, Zhang X, Clark E, Mulcahey M, Huang S, Shi YG (2010). TET1 is a DNA-binding protein that modulates DNA methylation and gene transcription via hydroxylation of 5-methylcytosine. Cell Research.

[20] He YF, Li B Z, Li Z, Liu P, Wang Y, Tang Q, Ding J, Jia Y, Chen Z, Li L, Sun Y, Li X, Dai Q, Song CX, Zhang K, He C, Xu GL (2011). Tet-mediated formation of 5-carboxylcytosine and its excision by TDG in mammalian DNA. Science.

[21] Ito S, Shen L, Dai Q, Wu SC, Collins LB, Swenberg JA, He C, Zhang Y (2011). Tet proteins can convert 5-methylcytosine to 5-formylcytosine and 5-carboxylcytosine. Science.

[22] Guo J U, Su Y, Zhong C, Ming GL, Song H (2011). Hydroxylation of 5-methylcytosine by TET1 promotes active DNA demethylation in the adult brain. Cell.

[23] Wu H, Zhang Y (2011). Mechanisms and functions of Tet protein-mediated 5-methylcytosine oxidation. Genes & Development.

[24] Cortellino S, Xu J, Sannai M, Moore R, Caretti E, Cigliano A, Le Coz M, Devarajan K, Wessels A, Soprano D, Abramowitz LK, Bartolomei MS, Rambow F, Bassi MR, Bruno T, Fanciulli M, Renner C, Klein-Szanto AJ, Matsumoto Y, Kobi D, Davidson I, Alberti C, Larue L, Bellacosa A (2011). Thymine DNA glycosylase is essential for active DNA demethylation by linked deamination-base excision repair. Cell.

[25] Popp C, Dean W, Feng S, Cokus S J, Andrews S, Pellegrini M, Jacobsen SE, Reik W (2010). Genome-wide erasure of DNA methylation in mouse primordial germ cells is affected by AID deficiency. Nature.

[26] Wu H, Zhang Y (2011). Tet1 and 5-hydroxymethylation: A genome-wide view in mouse embryonic stem cells. Cell Cycle.

[27] Nabel CS, Jia H, Ye Y, Shen L, Goldschmidt HL, Stivers JT, Zhang Y, Kohli RM (2012). AID/APOBEC deaminases disfavor modified cytosines implicated in DNA demethylation. Nat Chem Biol.

[28] Gu TP, Guo F, Yang H, Wu HP, Xu GF, Liu W, Xie ZG, Shi L, He X, Jin SG, Iqbal K, Shi YG, Deng Z, Szabo PE, Pfeifer GP, Li J, Xu GL (2011). The role of Tet3 DNA dioxygenase in epigenetic reprogramming by oocytes. Nature.

[29] Iqbal K, Jin SG, Pfeifer GP, Szabo PE (2011). Reprogramming of the paternal genome upon fertilization involves genome-wide oxidation of 5-methylcytosine. Proc Natl Acad Sci USA.

[30] Wossidlo M, Nakamura T, Lepikhov K, Marques CJ, Zakhartchenko V, Boiani M, Arand J, Nakano T, Reik W, Walter J (2011). 5-Hydroxymethylcytosine in the mammalian zygote is linked with epigenetic reprogramming. Nat Commun.

[31] Inoue A, Shen L, Dai Q, He C, Zhang Y (2011). Generation and replication-dependent dilution of 5fC and 5caC during mouse preimplantation development. Cell Research.

[32] Inoue A, Zhang Y (2011). Replication-dependent loss of 5-hydroxy methylcytosine in mouse preimplantation embryos. Science.

[33] Branco MR, Ficz G, Reik W (2012). Uncovering the role of 5-hydroxymethylcytosine in the epigenome. Nature Reviews Genetics.

[34] Koh KP, Yabuuchi A, Rao S, Huang Y, Cunniff K, Nardone J, Laiho A, Tahiliani M, Sommer CA, Mostos lavsky G, Lahesmaa R, Orkin SH, Rodig SJ, Daley GQ, Rao A (2011). Tet1 and Tet2 regulate 5-hydroxymethylcytosine production and cell lineage specification in mouse embryonic stem cells. Cell Stem Cell.

[35] Szwagierczak A, Bultmann S, Schmidt CS, Spada F, Leonhardt H (2010). Sensitive enzymatic quantification of 5-hydroxy methylcytosine in genomic DNA. Nucleic Acids Research.

[36] Dawlaty MM, Ganz K, Powell BE, Hu YC, Markoulaki S, Cheng AW, Gao Q, Kim J, Choi SW, Page DC, Jaenisch R (2011). Tet1 is dispensable for maintaining pluripotency and its loss is compatible with embryonic and postnatal development. Cell Stem Cell.

[37] Ficz G, Branco MR, Seisenberger S, Santos F, Krueger F, Hore TA, Marques CJ, Andrews S, Reik W (2011). Dynamic regulation of 5-hydroxymethylcytosine in mouse ES cells and during differentiation. Nature.

[38] Pastor WA, Pape UJ, Huang Y, Henderson HR, Lister R, Ko M, McLoughlin EM, Brudno Y, Mahapatra S, Kapranov P, Tahiliani M, Daley GQ, Liu XS, Ecker JR, Milos PM, Agarwal S, Rao A (2011). Genome-wide mapping of 5-hydroxy methylcytosine in embryonic stem cells. Nature.

[39] Stroud H, Feng S, Morey Kinney S, Pradhan S, Jacobsen SE (2011). 5-Hydroxymethylcytosine is associated with enhancers and gene bodies in human embryonic stem cells. Genome Biology.

[40] Szulwach KE, Li X, Li Y, Song CX, Han JW, Kim S, Namburi S, Hermetz K, Kim JJ, Rudd MK, Yoon YS, Ren B, He C, Jin P (2011). Integrating 5-hydroxymethylcytosine into the epigenomic landscape of human embryonic stem cells. PLoS Genetics.

[41] Williams K, Christensen J, Pedersen MT, Johansen JV, Cloos PA, Rappsilber J, Helin K (2011). TET1 and hydroxy methylcytosine in transcription and DNA methylation fidelity. Nature.

[42] Szulwach KE, Li X, Li Y, Song CX, Wu H, Dai Q, Irier H, Upadhyay AK, Gearing M, Levey AI, Vasanthakumar A, Godley LA, Chang Q, Cheng X, He C, Jin P (2011). 5-hmC-mediated epigenetic dynamics during postnatal neurodevelopment and aging. Nature Neuroscience.

[43] Song CX, Szulwach KE, Fu Y, Dai Q, Yi C, Li X, Li Y, Chen CH, Zhang W, Jian X, Wang J, Zhang L, Looney TJ, Zhang B, Godley LA, Hicks LM, Lahn BT, Jin P, He C (2011). Selective chemical labeling reveals the genome-wide distribution of 5-hydroxymethylcytosine. Nature Biotechnology.

[44] Jin SG, Kadam S, Pfeifer GP (2010). Examination of the specificity of DNA methylation profiling techniques towards 5-methylcytosine and 5-hydroxymethylcytosine. Nucleic Acids Research.

[45] Song CX, Clark TA, Lu XY, Kislyuk A, Dai Q, Turner SW, He C, Korlach J (2012). Sensitive and specific single-molecule sequencing of 5-hydroxymethylcytosine. Nature Methods.

[46] Yu M, Hon GC, Szulwach KE, Song CX, Zhang L, Kim A, Li X, Dai Q, Shen Y, Park B, Min JH, Jin P, Ren B, He C (2012). Base-Resolution Analysis of 5-Hydroxymethylcytosine in the Mammalian Genome. Cell.

[47] Booth MJ, Branco MR, Ficz G, Oxley D, Krueger F, Reik W, Balasubramanian S (2012). Quantitative sequencing of 5-methyl cytosine and 5-hydroxymethylcytosine at single-base resolution. Science.

[48] Weber M, Hellmann I, Stadler MB, Ramos L, Paabo S, Rebhan M, Schubeler D (2007). Distribution, silencing potential and evolutionary impact of promoter DNA methylation in the human genome. Nature Genetics.

[49] Takahashi K, Yamanaka S (2006). Induction of pluripotent stem cells from mouse embryonic and adult fibroblast cultures by defined factors. Cell.

[50] Doege CA, Inoue K, Yamashita T, Rhee DB, Travis S, Fujita R, Guarnieri P, Bhagat G, Vanti WB, Shih A, Levine RL, Nik S, Chen EI, Abeliovich A (2012). Early-stage epigenetic modification during somatic cell reprogramming by Parp1 and Tet2. Nature.

[51] Mikkelsen TS, Hanna J, Zhang X, Ku M, Wernig M, Schorderet P, Bernstein B E, Jaenisch R, Lander ES, Meissner A (2008). Dissecting direct reprogramming through integrative genomic analysis. Nature.

[52] Cao R, Zhang Y (2004). SUZ12 is required for both the histone methyltransferase activity and the silencing function of the EED-EZH2 complex. Mol Cell.

[53] Cao R, Zhang Y (2004). The functions of E(Z)/EZH2-mediated methylation of lysine 27 in histone H3. Curr Opin Genet Dev.

[54] Cao R, Tsukada Y, Zhang Y (2005). Role of Bmi-1 and Ring1A in H2A ubiquitylation and Hox gene silencing. Mol Cell.

[55] Wang H, Wang L, Erdjument-Bromage H, Vidal M, Tempst P, Jones RS, Zhang Y (2004). Role of histone H2A ubiquitination in Polycomb silencing. Nature.

[56] Czermin B, Melfi R, McCabe D, Seitz V, Imhof A, Pirrotta V (2002). Drosophila enhancer of Zeste/ESC complexes have a histone H3 methyltransferase activity that marks chromosomal Polycomb sites. Cell.

[57] Muller J, Hart CM, Francis NJ, Vargas ML, Sengupta A, Wild B, Miller EL, O'Connor MB, Kingston RE, Simon JA (2002). Histone methyltransferase activity of a Drosophila Polycomb group repressor complex. Cell.

[58] de Napoles M, Mermoud JE, Wakao R, Tang YA, Endoh M, Appanah R, Nesterova TB, Silva J, Otte AP, Vidal M, Koseki H, Brockdorff N (2004). Polycomb group proteins Ring1A/B link ubiquitylation of histone H2A to heritable gene silencing and X inactivation. Developmental Cell.

[59] Sparmann A, van Lohuizen M (2006). Polycomb silencers control cell fate, development and cancer. Nature Reviews Cancer.

[60] Valk-Lingbeek ME, Bruggeman SW, van Lohuizen M (2004). Stem cells and cancer, the polycomb connection. Cell.

[61] Spivakov M, Fisher AG (2007). Epigenetic signatures of stem-cell identity. Nature Reviews Genetics.

[62] Bracken AP, Dietrich N, Pasini D, Hansen KH, Helin K (2006). Genome-wide mapping of Polycomb target genes unravels their roles in cell fate transitions. Genes & Development.

[63] Boyer LA, Plath K, Zeitlinger J, Brambrink T, Medeiros LA, Lee TI, Levine SS, Wernig M, Tajonar A, Ray MK, Bell GW, Otte AP, Vidal M, Gifford DK, Young RA, Jaenisch R (2006). Polycomb complexes repress developmental regulators in murine embryonic stem cells. Nature.

[64] Lee TI, Jenner RG, Boyer LA, Guenther MG, Levine SS, Kumar RM, Chevalier B, Johnstone SE, Cole MF, Isono K, Koseki H, Fuchikami T, Abe K, Murray HL, Zucker JP, Yuan B, Bell GW, Herbolsheimer E, Hannett NM, Sun K, Odom DT, Otte AP, Volkert TL, Bartel DP, Melton DA, Gifford DK, Jaenisch R, Young RA (2006). Control of developmental regulators by Polycomb in human embryonic stem cells. Cell.

[65] Richly H, Aloia L, Di Croce L (2011). Roles of the Polycomb group proteins in stem cells and cancer. Cell Death Dis.

